# Mouse models of chronic lymphocytic leukemia and Richter transformation: what we have learnt and what we are missing

**DOI:** 10.3389/fimmu.2024.1376660

**Published:** 2024-06-06

**Authors:** Maria Teresa Sabrina Bertilaccio, Shih-Shih Chen

**Affiliations:** ^1^ Department of Experimental Therapeutics, The University of Texas MD Anderson Cancer Center, Houston, TX, United States; ^2^ Institute of Molecular Medicine, The Feinstein Institutes for Medical Research, Manhasset, NY, United States

**Keywords:** CLL, Richter transformation, mouse model, tumor microenvironment, CRISPR

## Abstract

Although the chronic lymphocytic leukemia (CLL) treatment landscape has changed dramatically, unmet clinical needs are emerging, as CLL in many patients does not respond, becomes resistant to treatment, relapses during treatment, or transforms into Richter. In the majority of cases, transformation evolves the original leukemia clone into a diffuse large B-cell lymphoma (DLBCL). Richter transformation (RT) represents a dreadful clinical challenge with limited therapeutic opportunities and scarce preclinical tools. CLL cells are well known to highly depend on survival signals provided by the tumor microenvironment (TME). These signals enhance the frequency of immunosuppressive cells with protumor function, including regulatory CD4^+^ T cells and tumor-associated macrophages. T cells, on the other hand, exhibit features of exhaustion and profound functional defects. Overall immune dysfunction and immunosuppression are common features of patients with CLL. The interaction between malignant cells and TME cells can occur during different phases of CLL development and transformation. A better understanding of *in vivo* CLL and RT biology and the availability of adequate mouse models that faithfully recapitulate the progression of CLL and RT within their microenvironments are “*conditio sine qua non”* to develop successful therapeutic strategies. In this review, we describe the xenograft and genetic-engineered mouse models of CLL and RT, how they helped to elucidate the pathophysiology of the disease progression and transformation, and how they have been and might be instrumental in developing innovative therapeutic approaches to finally eradicate these malignancies.

## Introduction

**Figure 1:**
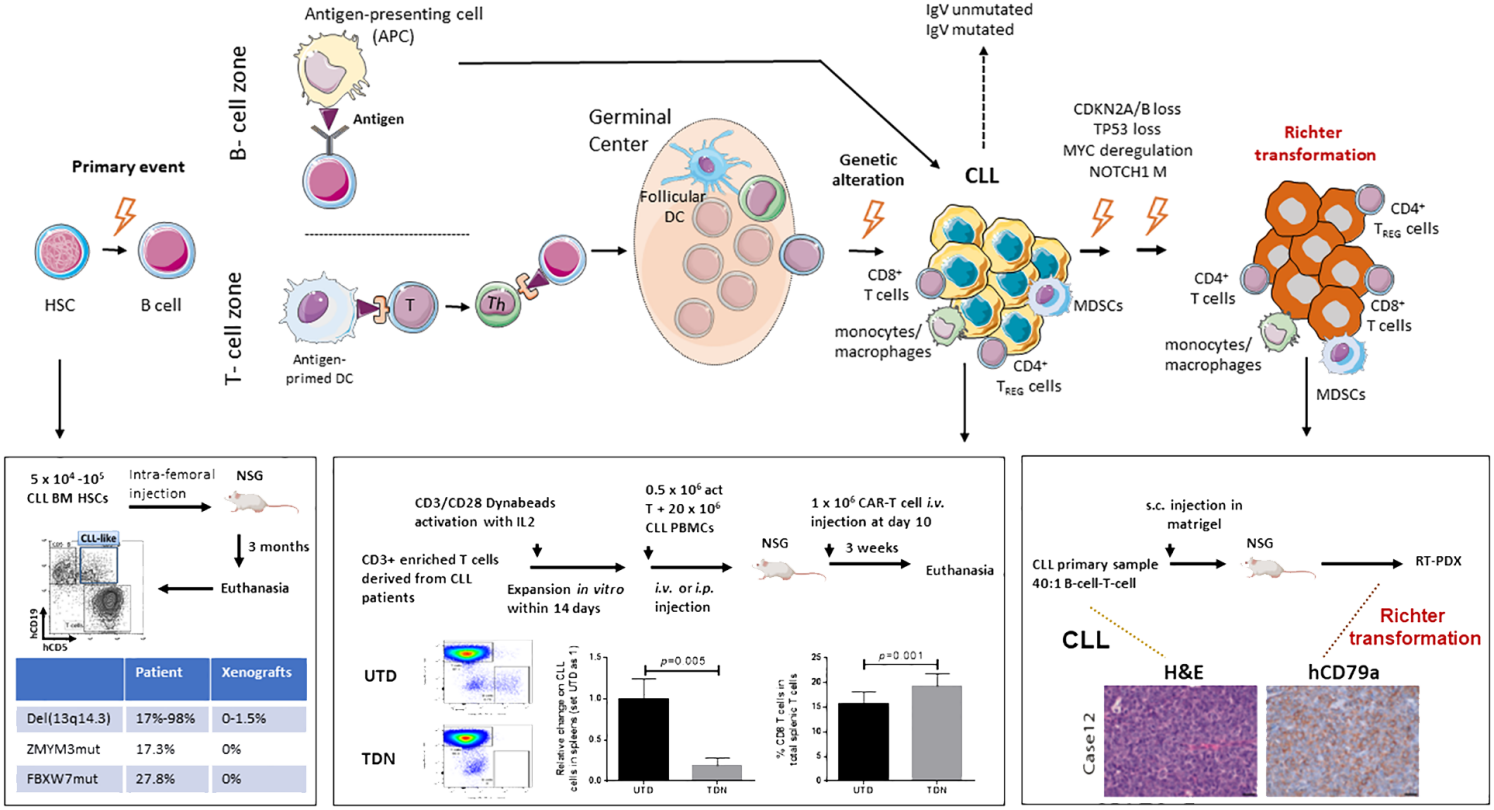
Schematic model of CLL initiation, progression, and Richter transformation. Initial genetic events occur during the B-cell development at the stage of hematopoietic stem cells HSC (1–3). Additional genetic abnormalities, within either a T-cell-dependent or -independent phase, then trigger the progression into CLL (4, 5). Finally, through the acquisition of independent genetic events, Richter transformation (RT) occurs (6–9). A complex network of reactive cells of the immune microenvironment plays a critical role within every phase of the biology of the disease, before and after transformation, either in the lymphoid tissues or peripheral blood (9). HSC, hematopoietic stem cell; DC, dendritic cell; MDSCs, myeloid-derived suppressor cells; T_REG_, CD4^+^regulatory T cells; Th, CD4^+^ helper T cells. Published datasets are from Patten et al. (10), Chiang et al. (11), and Playa-Albinyana et al. (8).

Chronic lymphocytic leukemia (CLL) is a disease with remarkable complexity that can evolve into Richter transformation (RT), an aggressive lymphoma with a dismal prognosis (**Figure 1**). CLL cells are enriched not only with old/quiescent cells, but also with a small fraction of recently born/proliferating cells. Division of CLL cells mainly occurs in lymph nodes (LNs) but not in the bone marrow or blood (12), highlighting the importance of the tumor microenvironment (TME) in the pathophysiology of this malignancy. CLL is also a disease with genetic complexity; CLL patients have common mutations involved in driving disease progression (13–15) and RT (6, 7, 16, 17). These genetic abnormalities are already present in CLL patient bone marrow CD34^+^ hematopoietic stem cells (HSCs) (1, 18, 19) and in the pre-leukemic stage of monoclonal B-cell lymphocytosis (MBL) (20–22). In contrast, the nonmalignant immune cells within TME revealed transcriptional similarity across patients (21, 23).

Developing mouse models faithfully mimicking CLL would facilitate the understanding of disease mechanisms, especially those driven by the crosstalk between the tumor and the TME. Preclinical mouse models that closely represent patient disease are also indispensable to improve treatments. Here, we review the recently developed genetic-engineered (GEMMs) and patient-derived xenograft (PDX) mouse models of CLL and RT.

GEMMs have contributed significantly to the field of CLL research. With the recently developed CRISPR-Cas9 technique, multiplexed-GEMMs have been established (11, 24–28). These GEMMs capturing driver mutations of CLL develop *de novo* tumors. Tumors arising from multiplexed-GEMM mice closely mimic the genetic heterogeneity of their human counterparts (11, 24) and are capable of spontaneously transforming into RT (26–28). Because GEMMs capture both extrinsic factors from TME and the intrinsic properties of CLL, these mice are suitable for *in vivo* validation of candidate cancer-driven genes and therapeutic agents targeting the crosstalk between tumor and TME. However, the current GEMMs still have drawbacks; for example, none of the GEMMs of CLL recapitulate the development of IgHV-mutated versus unmutated CLL, or are capable of modeling responses to existing treatment history in CLL patients.

The usefulness of PDXs in studying CLL and RT depends on the level of relatedness of the disease characteristics between these models and patients. There are several features of xenografts to consider for the successful translation into clinics. First, they must faithfully recapitulate the spectrum and the heterogeneity of lymphoproliferation observed in patients. Second, xenografts should have the genetic, phenotypic, and clinical features of the human disease. Third, all relevant CLL and RT events occur in permissive tissue microenvironments, and xenograft systems must fully mimic the co-evolution of malignant clones with nonmalignant cell types. This is especially a problem with PDXs that require serial adoptive transfers after the first inoculation. PDX capture clonal selection and evolution in an immunodeficient murine microenvironment that do not reflect human counterparts. Thus, the reliability of such models has been hampered in the past by the availability of proper humanized recipient mice able to fully reconstitute the human tumor immune microenvironment.

## Mouse models of CLL

### Xenograft models of CLL patient-derived bone marrow CD34+ hematopoietic stem cells

Accumulated evidence suggests that CLL is a stepwise disease, preceded by a pre-leukemic state. Driver mutations such as *SF3B1* commonly seen in CLL tumors are also present in CLL patient bone marrow CD34^+^CD19^-^ HSCs (1, 18, 19, 29) and MBL patient B cells (22, 30, 31). Functionally, BM-HSCs from both early- and late-stage CLL patients display increased protein levels of HIF-1a, GATA-1, PU.1, and GATA-2, and are poorly responsive to colony-forming unit (CFU) assays (2). In 2011, Kikushige et al. (3) injected CLL patient BM-HSCs in NOD-SCID/IL-2Rγ^null^ (NSG) and NOD-Rag1^null^IL2rγ^null^ (NRG) mice (**Table 1**) and found the CLL-like mono- or oligo-clonal B cells in the recipients; however, B cells implanted in mice were not clonally related to the original patient cells. Similarly, in 2022, Chiang et al. intra-femorally injected CLL-BM HSCs in busulfan pre-conditioned NSG xenografts (11) and obtained CLL-like cells with VDJ rearrangements distinct from those of the originally transplanted CLL patient cells. Notably, the renewal and survival of HSCs were dependent on GATA2 and IKZF2 (11). Altogether, current xenografts of CLL BM-HSCs support the differentiation of CLL-like B cells that are clonal unrelated to patients, and none of the mice develop a full-blown CLL disease (**Figure 1**).

**Table 1:** Xenograft models of CLL and RT.

Model	Description	Reference
Xenograft models of CLL patient-derived CD34^+^ hematopoietic stem cells (HSC)		
NOD-SCID/IL-2Rγ^null^ (NSG)	Transplantation of bone marrow-derived HSCs into newborn mice (facial vein injection)	Kikushige Y (3)
NOD-SCID/IL-2Rγ^null^ (NSG)	Transplantation of bone marrow-derived HSCs in busulfan pre-conditioned xenograft (intra-femoral injection)	Chiang CL (11)
Xenograft models of primary CLL patient cells		
Human/mouse chimera	Transplantation of CLL PBMCs into irradiated BALB/c mice pre-conditioned with SCID mouse bone marrow	Shimoni A (32)
NOD/SCID	Transplantation of CLL PBMCs into NOD/SCID mice	Durig J (33)
NOD-SCID/IL-2Rγ^null^ (NSG)	Co-transfer of CLL PBMCs with allogeneic APCs (CD14^+^ or CD19^+^ cells)	Bagnara D (34)
NOD-SCID/IL-2Rγ^null^ (NSG)	Co-transfer of CLL PBMCs with pre-activated autologous T cells	Patten PE (10, 35)
Patient-derived xenograft (PDX) models of Richter transformation		
NOD-SCID/IL-2Rγ^null^ (NSG)	Subcutaneous injection of RT patient-derived lymph node cell suspensions with matrigel	Vaisitti T (36)
NOD-SCID/IL-2Rγ^null^ (NSG)	Subcutaneous injection of RT patient-derived lymph node cell suspensions with matrigel	Fiskus W (27)
NOD-SCID/IL-2Rγ^null^ (NSG)	Subcutaneous injection of RT patient-derived lymph node cell suspensions with matrigel	Vaisitti T (37)
NOD-SCID/IL-2Rγ^null^ (NSG)	Co-transfer of B and T cells from the peripheral blood of a CLL patient known to undergo transformation into clonally related Richter	Playa-Albinyana H (8)

### Xenograft models of primary CLL patient cells

The first xenografts using primary CLL patient cells were established by Berrebi and Reisner (32, 38) using irradiated BALB/c mice pre-conditioned with SCID mouse bone marrow (**Table 1**), followed by Dürig et al. using NOD/SCID mice intraperitoneally injected with primary tumors obtained from CLL patient blood mice (33, 39). Both studies evidentiated a disease-stage-dependent CLL cell engraftment; PBMCs from late-stage patients consistently engraft better in mice. Similar results were also observed when Chiorazzi’s group inoculated CLL PBMCs together with autologous T cells in nonobese diabetes/severe combined immunodeficiency/γc (null) mice (34). This model was the first to report the reproducible engraftment of CLL cells in the mice and uncover the requirement of autologous T cells for the growth of CLL B cells (34). Chiorazzi’ s group further modified the model by injecting NSG mice with pre-activated autologous T cells and CLL-PBMCs at the ratio of 1:40, and again discovered T-dependent CLL B-cell proliferation in murine spleens (10, 35). CLL B cells were present in mouse spleens, but the percentage of CLL B cells was decreasing over time. In contrast, T-cell population increased and became the major population of total human lymphocytes (10). The predominant T cells but not CLL B cells at the late stage occurred even in mice injected with CLL PBMCs without pre-activated T cells (10).

Similar results were also shown by Wiestners’ group when they inoculated 60 million CLL PBMCs in NSG mice; again, none of the mice died from CLL (40, 41). However, CLL B cells engrafted in murine spleens highly resembled their original donor LN counterparts for the gene expression profiles, BCR, and NF-kB signal signatures (40, 41).

The feature of T cell-dependent CLL cell growth in NSG xenografts allows one to test novel therapies in the context of CLL B cell–T cell interaction. Clinically, anti-CD19 chimeric antigen receptor (CD19.CAR) T cells reject CLL tumors by overcoming immunological tolerance; however, the efficiency is low compared to other B-cell malignancies (42, 43). CD19. CAR T cells therefore have been tested in NSG-xenografts (42–44). CD19.CAR T cells (TDN) and control untransduced T cells generated from PBMCs obtained from three treatment-naive CLL patients were injected intravenously into NSG xenografts. Compared to the untransduced T-cell cohort, CD19.CAR T cell-treated mice have significantly increased the percentage of CD8 T cells and reduced CLL B cells in the spleens, suggesting that the model is suitable for developing strategies to improve the efficacy of CARs in CLL (**Figure 1**).

### Genetic-engineered mouse models of CLL for studies in TME

Different from xenografts, GEMMs of CLL allow the preclinical TME intervention studies. GEMMs of CLL have shown the critical functions of non-malignant cells such as T cells including Foxp3^+^ CD4^+^ T-regulatory cells (T_REG_), monocytes/macrophages, dendritic cells, and stromal cells, including specialized antigen-presenting cells and follicular dendritic cells (FDCs) (**Figure 1**). Majority of these studies are based on *Eμ-TCL1* transgenic mouse model (**Table 2**), the mostly utilized GEMM for CLL, characterized by overexpression of human *TCL1* specifically in B cells (45). *TCL1* oncogene is common in CLL patients (55). These mice develop aggressive disease similar to IGHV-unmutated patients. Importantly, the malignant TCL1 CLL B cells are serial transferrable, allowing one to identify key factors within TME that impact CLL disease progression (56).

**Table 2:** Genetic-engineered mouse models (GEMMs) of CLL.

Model	Description		Reference
*Eμ-TCL1* transgenic (tg) mice	Exogenous expression of the human TCL1 oncogene under the control of the IGHV promoter and IGH enhancer (*Eμ*) results in the clonal expansion of CD5^+^ IgM^+^ B cells with unmutated IGHV genes, stereotypic IGHV and IGLV genes.		Bichi R (45)
*minimal deleted region (MDR)^−/−^* mice	*MDR^−/−^* mice lack mir-15a/16–1, dleu2 and dleu5 genes and develop MBL, CLL, and CD5^-^ NHLs. MDR*^−/−^* mice develop CLL with 22% penetrance and unmutated and stereotypic IGHV genes.		Klein U (46)
*mir-15a/16–1^−/−^* mice	Genetic inactivation of *mir-15a and mir-16–1* in mice results in the development of MBL, CLL, and NHLs. *mir-15a/16–1^−/−^* mice develop CLL with 20% penetrance and unmutated and stereotypic IGHV genes.		Lia M (47)
Model	Description	TME key findings	Reference
*Eμ-TCL1* tg adoptive transfer model	Investigation of the epigenetic and functional consequences of antigen-specific T-cell responses by transplanting OT-I CD8^+^ T cells in the *Eμ-TCL1* adoptive transfer model.	Impairment of CD8^+^ T-cell responses through epigenetic reprogramming	Martens AWJ (48)
*Eμ-TCL1* tg mice	Changes in regulatory T-cell phenotype and related expansion at different stages of leukemia have been evaluated in the *Eμ-TCL1* tg mice	Role of regulatory T cells in CLL progression	Goral A (49)
*Eμ-TCL1* tg adoptive transfer model	The *Eμ-TCL1* adoptive transfer model has been utilized to evaluate the interrelation between regulatory T cells and neutrophils in the CLL TME.	Immunosuppressive role of regulatory T cells and neutrophils in CLL	Goral A (50)
*Eμ-TCL1* tg adoptive transfer model	The *Eμ-TCL1* adoptive transfer model was instrumental to demonstrate that macrophage targeting via CSF1R blockade sensitizes leukemic cells to apoptosis and significantly impacts the whole TME	Role of macrophages and related targeting strategies in leukemia progression	Galletti G (51)
*Eμ-TCL1* tg mice	Skewing of myeloid cell populations with CLL development was documented in the *Eμ-TCL1* tg mice with particular focus on the monocytes and protumor macrophages	Role of patrolling CLL-associated monocytes and macrophages and related depletion in disease development	Hanna BS (52)
*Eμ-TCL1* tg mice	CXCR5-controlled access to follicular dendritic cells (FDCs) confers proliferative stimuli to CLL cells in the *Eμ-TCL1* tg mice	The role of FDCs in leukemia B-cell activation and proliferation	Heinig K (53)
*Eμ-TCL1* tg mice and *Eμ-TCL1* tg adoptive transfer model	The *Eμ-TCL1* tg mice and the *Eμ-TCL1* adoptive transfer model allowed researchers to characterize the evolution of the stromal microenvironment during CLL progression and to identify the involvement of the retinoid signaling	The role of the retinoid-signaling in leukemia-stroma crosstalk and CLL progression	Farinello D (54)

CLL disease is known to have dysfunctional immunity due to impaired activities of myeloid cells, neutrophils, dendritic cells, and T cells. CLL cells impact non-malignant supporting cells to alter their functions and phenotype in favor of leukemic growth. Recently, Martens et al. explored the antigen-specific response of naïve OT-I CD8^+^ T cells to antigen mCMV-OVA and showed that TCL1 leukemic B cells induced epigenetic modifications and skewing of short-lived effector cells in these antigen-specific T cells (48). CLL infiltration also alters the subsets of T cells. Using the TCL1 mouse system (49), Goral et al. discovered impacted neutrophils and T_REG_ including the subset of CD44^low^CD25^low^ T_REG_ after CLL B-cell infiltration; CLL tumors activate T_REG_ to block CD62L and IL-4 receptor expressed on neutrophils, and further suppress neutrophil functions. Depletion of T_REG_ cells restores the impaired neutrophils and induces changes in the CLL TME (50). In TCL1 mice, CLL cells initially accumulate in the peritoneal cavity; this also triggers the infiltration of monocytes and macrophages mainly expressing protumor signature, including CD206, CD124, and ARG-1 molecules in the peritoneum. At later stages, when CLL cells accumulate in the enlarged spleens, patrolling monocytes expressing high levels of PD-L1 were found accumulating in spleens (52). Targeting macrophages sensitizes CLL to apoptosis and delays disease progression (51).

Stromal cells clearly regulate the dynamic behavior of CLL cells, contributing to homing and trafficking in and out of the tissues, even during treatment. In 2014, Heinig et al. (53) demonstrated the key function of FDCs in the *Eμ-TCL1* transgenic mouse model. Heinig et al. (53) knocked out CXCR5 in TCL1 CLL B cells and uncovered the CXCR5-regulated access of CLL cells to FDCs; CXCR5-expressing CLL cells further stimulate CXCL13 secretion and stromal cell remodeling. In 2018, Farinello et al. (54) discovered that TCL1 CLL B cells induce CXCL13 expression in the remodeled stromal microenvironment; this process is dependent on the induction of retinoid (RA) signaling in stromal cells; targeting RA signaling delays disease progression and prolongs overall survival. Consistent with these observations, the expression of RA nuclear receptors (54) and plasma levels of CXCL13 (57) correlates with bad prognosis in CLL patients.

Bone marrow niche is the site where CLL malignancy begins with primary genetic mutations followed by antigen-driven expansion (58, 59). The TME of BM is known to contribute not only to the survival of malignant cells (60), but also to the development of drug resistance (61, 62). In CLL, the BM infiltration of CLL cells causes the bone erosion and thinning of the femoral cortex in a xenograft NSG mouse model via the activation of the RANK/RANKL signaling (63). The BM environmental RANKL-RANK signaling provides the survival of CLL cells, shown by Alankus et al. in mice that express hyperactive RANK^k240E^ transgenic gene in B lymphocytes; *ex vivo*, RANKL-expressed BM stromal cells also support the survival and proliferation of TCL-1 murine CLL cells and MEC-1 cells (64). Although the potential effects of anti-RANKL in counteracting chemoresistance or targeted therapy resistance has not been tested, the contact of CLL cells and stromal cells is known to lead to drug resistance (65, 66). Thus, modulation of the BM microenvironment might provide opportunities to improve treatment outcome. However, a suitable animal experimental model that can recapitulate the significance of BM TME in CLL patients is still lacking.

Besides TCL1 oncogene, deletion of *13q14* (del13q14) is the most frequent genetic lesion in CLL; 60% of CLL patients carry *del13q14*. The 13q14 region encodes genes highly conserved in human and mice; the minimal deleted region (MDR) includes the *DLEU2* long non-coding RNA (ncRNA), and the *miR-15a/miR-16–1* cluster. Klein et al. elegantly recapitulated the 13q14 deletion and CLL phenotype in MDR (46) and miR-15a/16–1-deleted mice (47). In 2023, Ten Hacken et al. created del(13q)-Cd19-Cas9 LSK cells, introduced control guide RNAs, and demonstrated CLL development already in mice carrying only del(13q)-B cells (26). In contrast, the generation of single loss-of-function (LOF) lesion using sgRNA targeting *Atm*, *Tp53*, *Birc3*, *Chd2*, *Mga*, or *Samhd1* was not sufficient to drive CLL disease development (25).

## Mouse models of Richter transformation

### Xenograft models of Richter transformation

The impact of the TME in CLL progression is more evident when the disease transforms into RT with dramatic LN involvement. RT is characterized by an evolution of CLL into an aggressive lymphoma. Two percent to 10% of patients with CLL develop diffuse large B-cell lymphoma (DLBCL)-RT with a median overall survival of less than 12 months (67). The whole genome, epigenome, and transcriptome of patient-derived RT cells have been extensively investigated by several independent groups. New driver alterations and a B-cell receptor (BCR)^LOW^-signaling transcriptional axis in RT cells have been identified (6, 7, 68, 69). Targeted therapies have not shown good responses in RT. Though CD19.CAR-T cell therapy is an established treatment for *de novo* DLBCL (70–72), data on the efficacy of CD19.CAR-T cell therapy in RT are limited and need better investigation (73, 74). High levels of PD1/PD-L1 checkpoint molecules have been observed on selected immune cells and encouraging results recently came from a phase 2 trial based on the combination of nivolumab and ibrutinib with an overall response rate of 42% (75). The evidence that checkpoint inhibitors show clinical activity in patients with RT compared to patients with CLL highlights the critical difference in the TME between RT and CLL that should be better investigated to improve outcomes in patients. Distinct immune signatures have been described in CLL and RT (76, 77). Patients with RT show a more diverse T-cell repertoire, lower T-cell TCR clonality, and increased infiltration of T_REG_ cells compared to patients with CLL (76). CD68^+^CD163^+^ protumor macrophages have been found at increased levels in LN sections of patients with RT compared to CLL (76).

The interaction between malignant cells and TME can occur during different phases of CLL progression and RT (**Figure 1**). How and when selected immune cells become dysfunctional and acquire a protumor phenotype during leukemia progression and whether this phenotype is exacerbated in patients undergoing RT is unexplored.

The availability of mouse models recapitulating the human RT with a fully reconstituted immune microenvironment is crucial to identify and preclinically develop therapeutic strategies for these uncurable malignancy.

To date, two PDX models of RT have been established in NSG mice (**Table 1**) by two independent groups (27, 36). Vaisitti et al. reported for the first time the development of two PDX models of RT documenting extensive involvement of the spleen (SP), bone marrow (BM), peripheral blood (PB), and extra-nodal organs (36). LN cell suspensions were injected subcutaneously with matrigel, and after the first engraftment, tumor cells were retransplanted *in vivo* for at least 10 passages to stabilize the PDX models. These models preserved the phenotypes, and the genomic and biomolecular features of the original RT in the patients. Targeted deep sequencing, whole-exome sequencing (WES), and RNA sequencing were exploited to characterize the two models that appeared to share 80% of their transcriptome with the original patient samples. Of note, one of the models maintained *in vivo* the *BTK* mutation associated to ibrutinib resistance (p.C481S) found in the original primary sample. Primary and PDX samples shared the same IGHV gene mutational status and were EBV negative, thus ruling out the possibility of EBV-driven non-malignant B-cell proliferation *in vivo*.

Three additional DLBCL-RT PDX models (HPRT1, HPRT2, and HPRT3) with similar pathophysiology features have been reported more recently in NGS mice (27). Immunoglobulin gene analysis performed on the PDX and the original samples allowed one to identify clonally related or unrelated models; the HPRT3 model was documented as clonally related to the original CLL/RT patient-derived sample, while HPRT2 was defined as clonally unrelated. Additionally, based on a detailed phenotypic characterization, two out of three PDX lines were identified as ABC-DLBCL type due to the expression of MUM/IRF4. The HPRT1 PDX line was described as GCB-DLBCL type expressing high levels of CD10 and BCL6. The RT-DLBCL cells were found growing in the BM, SP, and liver with marked splenomegaly and hepatomegaly (27). These RT-PDXs were found to display active enhancers, and protein expression of IRF4, TCF4, and BCL2, together with high sensitivity to BET inhibitors. Unlike RS9737 and RS13160, HPRT PDXs have been stabilized as cell lines for *in vitro* cytotoxicity studies. When exploited in survival experiments, these HPRT PDX models allowed one to preclinically test and demonstrate the activity of the combination based on BET-PROTAC and venetoclax, thus uncovering the potentiality of a novel treatment for patients with RT (27).

Two additional PDX models (RS1050 and IP867/17) have been reported by Valsitti et al. (37) using the same experimental strategy (36). IP867/17 was developed from an untreated patient. These PDX models were evaluated in flow cytometry for the expression of the Receptor tyrosine kinase-like orphan receptor 1 (ROR1), a known tumor-specific target (78). The antibody–drug conjugate VLS-101 combining the ROR1 targeting moiety and monomethyl auristatin E (MMAE) has been preclinically tested in these PDX models and showed a favorable impact on the *in vivo* growth and survival of RS PDX models (37). These results performed in the RT PDX models supported the development of the phase 1 trial NCT03833180 in patients with RT. Of note, further preclinical studies in the RT PDX models RS1316 and IP867/17 helped demonstrate *in vivo* the synergistic effect of the dual phosphatidylinositol 3-kinase-d/g (PI3K-d/g) inhibitor duvelisib and the Bcl-2 inhibitor venetoclax and allowed the enrollment of patients with RT in the trial NCT03892044 combining the two agents. More recently, Deaglio’s group preclinically evaluated the targeting of the surface antigen CD37 in the all the PDX models developed by her group (79). Three amanitin-based ADC anti-CD37 agents were tested in four established RT PDX models and significantly prolonged the mice survival.

Very recently, Playa-Albinyana H et al. generated an RT-PDX model (Case 12, **Figure 1**) mimicking the evolution of CLL into RT by injecting B cells and T cells from the peripheral blood of a patient with CLL, known to undergo transformation into clonally related Richter 20 years later after ibrutinib treatment (8). An additional RT-PDX was developed by the same group by transplanting B cells and T cells from a patient with RT (Case 19). Of note, they characterized over time *in vivo* the dynamics of the subclonal architecture and identified in the xenotransplanted mice the engraftment of a small subclone originally present in the patient RT19 that acquired later in the mice relevant alterations including *BCL2* and *MYC* (8). As in the previous PDX models, they confirmed in the mice the RT transcriptional profile. This study confirms the concept of early seeding of RT subclones in the circulation of patients with CLL and elegantly described *in vivo* the evolutionary process of transformation (7).

Overall, these models (27, 36) maintain the malignant phenotype, genomic architecture, and biomolecular signature of the original tumors and have been successfully exploited *in vivo* and *in vitro* to preclinically test the activity of new agents. However, they do not recapitulate LN dissemination, which is a typical feature of RT in patients in the context of a fully immune reconstituted patient-derived microenvironment.

### Genetically engineered mouse models of Richter transformation

In 1992, the first mouse model of RT was documented by E.S. Raveche and her group within the context of the NZB mouse strain (**Table 3**). Multiple passages through successive F1 recipients of a clonal line originating from an old NZB mouse resulted in a transformed clone localizing to the LNs and liver with the distinct features of the human RT (80, 88, 89). Unlike the original CLL-like clone, the murine secondary transformation recapitulated the pathology of RT with the disruption of the normal tissue architecture and the massive infiltration of the spleen, LNs, and liver by large cells with cleaved nuclei and evident nucleoli (88).

**Table 3:** Genetic-engineered mouse models (GEMMs) of RT.

Model	Description and key findings	Reference
B-1 line originating from NZB mice	During serial passages, an aggressive Richter-like lymphoma developed as a result of transformation from the original B-1 CLL clone	Peng B (80)
*Eμ-TCL1Trp53^−/−^* mice	*Eμ-TCL1* mice with conditional B-cell specific deletion of Trp53 display occasional transformation into Richter	Knittel G (81)
*Eμ-TCL1xMyc* mice	*Eμ-TCL1* were crossed with *Eμ-Myc* mice to investigate the clinical phenotype associated with the expression of these oncogenes. The mice developed features of aggressive lymphoma including Richter transformation	Lucas F (82)
*Eμ-TCL1 Nfat2^−/−^*	Deletion of Nfat2 in the context of the *Eμ-TCL1* tg mouse results in the development of Richter-like phenotype	Muller DJ (83)
*Eμ-TCL1* LCK*^−/−^*	*Eμ-TCL1* tg mice with genetic loos of LCK show acceleration of CLL with RT-like features	Marklin M (84)
*Eμ-TCL1^Akt-C^*	Genetic overactivation of Akt in the *Eμ-TCL1* mouse model results into transformation of CLL into RT with reduced survival and aggressive lymphoma phenotype.	Kolhaas V (85)
Inactivation of CDKN2A, CDKN2B, and TP53 in the *Eμ-TCL1* tg adoptive transfer model	Simultaneous disruption of CDKN2A, CDKN2B, and TP53 in the *Eμ-TCL1* tg-derived cells leads to aggressive disease with RT features.	Chakraborty S (87).
*Eμ-TCL1/PRMT5*	*Eμ-TCL1* tg mice with overexpression of hPRTMT5 develop highly aggressive lymphoma with histological features of RT.	Hing ZA (69)
*MGA^−/−^ MDR^−/−^ Sf3b1^mut^*	Deletion of MGA in the *MDR^−/−^ Sf3b1^mut^* CLL mouse model leads to a mouse model of RT, where cells exhibit mitochondrial aberrations with elevated oxidative phosphorylation (OXPHOS)	Iyer (91)
Multiplexed *in vivo* CRISPR-Cas9 B cell editing of LOF in *ATM*,*TP53*, *CHD2*, *BIRC3*, *MGA*, *SAMHD1*, combined with *del*(13q)	Modeling the genetic heterogeneity of CLL through multiplexed *in vivo* CRISPR-Cas9 B cell editing of recurrent CLL loss of function drivers, recapitulates the transformation of CLL into Richter	Ten Hacken E (26)

Almost two decades later, two TCL1-driven models of high-risk CLL have been generated with the conditional B-cell-specific deletion of Tp53 that displayed occasionally the features of RT with the occurrence of large CD5^-^ blastoid cells in the splenic infiltrates (81).

Then, a mouse model resembling RT, the double transgenic Eµ-TCL1xMyc, has been reported with features of concurrent CLL and highly aggressive lymphoma (82). This model was exploited in preclinical studies to test the BTK inhibitor ARQ531 and helped demonstrate its superior activity over ibrutinib in survival experiments (90).

More recently, the B-cell-specific deletion of either the transcription factor NFAT2 (83) or its target gene tyrosine kinase LCK (84) in the TCL1 transgenic mice was shown to induce the acceleration of CLL and the development of an RT-like phenotype.

RT has been associated with somatic mutations involving TP53, CDKN2, MYC, EGR2, and NOTCH1. Kohlhaas et al. demonstrated that high levels of AKT phosphorylation occur in patients with high-risk CLL and RT with TP53 and NOTCH1 mutations (85). The genetic inactivation of Akt in the TCL1 transgenic mice led to the development of a typical RT phenotype, with mice carrying splenomegaly, emerging large blastoid cells with pleomorphic nuclei, and high levels of lactate dehydrogenase (85). Of note, Kohlhaas et al. showed that Akt-mediated control promotes cell–cell interaction, the induction of CD4^+^ T cells, and the overexpression of DII1, which induces NOTCH1 activation and facilitates RT transformation. Overall, this model helped to demonstrate that the potential inhibition of PI3K/AKT and NOTCH1 might be a strategy to explore patients with high-risk CLL and RT. This model validates several evidence observed in RT PDX models and an ongoing multicenter trial with the PI3Kδ,γ inhibitor duvelisib (86).

Additionally, an interesting model has been reported by the group of D. Efremov (87). Unlike the above-described models, they mimicked in mice for the first time multiple genetic lesions associated to RT, thus better recapitulating the genetic evolution of the disease. By using the (CRISPR)/Cas9 technology, they demonstrated that the simultaneous inactivation of CDKN2A, CDKN2B, and TP53 in primary TCL1 transgenic-derived murine CLL cells induces proliferation *in vitro* and accelerates tumor growth in the TCL1 tg transplantation system. The administration of BCR and CDK4/6 inhibitors ibrutinib and palbociclib has a favorable impact on the survival of mice transplanted with the CLL murine cells carrying the CDKN2A, CDKN2B, and TP53 lesions (87). These data gave relevant indications on the treatment of a subset of RT patients with TP53 and CDKN2A/2B abnormalities, suggesting the combination of BCR inhibitor with CDK4/6 inhibitors such as palbociclib. Overall, this evidence highlights the importance of simultaneously mimicking *in vivo* the genetic lesions observed in distinct subsets of patients with RT to investigate the activity of new combination agents.

PRMT5 is known to regulate oncogenes such as NOTHC1, c-MYC, and P53 that are often dysregulated in patients with RT. Recently, Hing et al. demonstrated that PRMT5 is expressed in patients with RT transformation leading to the hypothesis that it might be involved in the transformation (68). Indeed, they generated PRMT5/TCL1 double transgenic mice developing an aggressive lymphoma with the clinical features of RT, including lymphadenopathy and palpable splenomegaly (68).

Together with TP53, CDKN2A/B deletions, and NOTCH activations, additional genetic lesions have been identified in patients with RT, including the loss-of-function mutations and deletions in Max gene associated (MGA), a MYC transcriptional repressor (7). Iyer et al. established a new model of RT by knocking out Mga in an Sf3b1/Mdr model of CLL (91). In detail, they crossed the murine CLL line CD19^cre/+^Mdr^fl/+^Sf3b1 K700E^fl/+^ with a mouse strain that conditionally expresses Cas9 to obtain a donor mouse line Cd19-Cre^fl/+^Sf3b1^fl/+^ Mdr^fl/+^Cas9^fl/+^. Murine hematopoietic stem cells, Lin^-^cKit^+^Sca1^+^ cells (CD45.2^+^), were then isolated from these mice and transduced *in vitro* with lentivirus expressing single guide RNA (sgRNA) targeting Mga. Edited cells were then transplanted into CD45.1^+^ recipient mice. When total splenic cells were secondarily transplanted into CD45.1^+^ recipient mice, rapid expansion of B220^+^ cells with CD5 loss and lymphoid tissue infiltration was observed. Cells became larger and acquired the morphology and phenotype of a more aggressive lymphoma with high proliferation index and expression of CD21 and CD71. Based on the immunoglobulin gene analysis, the CLL-like and RT cells were clonal. Further characterization of this model led to the identification of the MGA-NME1 axis as a driver of RT through the OXPHOS upregulation and uncovered a potential new targeting opportunity for patients with RT based on the simultaneous targeting of MYC and OXPHOS pathways (91).

Very recently, a sophisticated way to model *in vivo* the genetic complexity of CLL transformation into RT has been reported by Ten Hacken et al. via the multiplexed introduction of well-known loss-of-function CLL driver mutations (including *Atm, Trp53, Samhd1, Mga, Birc3*, and *Chd2*) into *del*(13q) murine B cells (26). Essentially, Lin^-^ cKit^+^ Sca1^+^ cells from donor mice expressing homozygous *del*(13q) were lentivirally transduced with sgRNA targeting six or five loss-of-functions lesions. Trp53 was present or absent in the multiplex to evaluate Trp53 involvement in the transformation. Transduced cells were then transplanted into either immunocompetent or immunodeficient NSG mice. CLL and RT lymphomas were observed either in immunocompetent or NSG mice; however, RT arose mainly in CD45.1^+^ recipient mice compared to NSG mice. All features of human RT histology were confirmed in murine RT. Further analyses allowed one to identify the co-occurrence of Trp53, Mga, and Chd2 lesions in RT and a tonic PI3K signaling as a characteristic feature of RT. Overall, this approach offers an interesting opportunity to model complex disease phenotypes and opens new venues of preclinical testing in uncurable malignancies (26).

## Conclusion

The xenograft models of primary CLL BM-HSCs or CLL PBMCs never gave a full-blown CLL disease, suggesting that additional genetic editing might be required. Recurrent mutations such as *NOTCH1*, *MYC, SF3B1*, *BRAF*, *TP53*, *XPO1*, *MED12*, *NFKBIE*, and *EGR2* are commonly seen in various subsets of CLL patients (14, 29, 92–95). However, because of the technique limitation to transfect primary CLL patient BM-HSCs or primary CLL patient B cells, modeling the driver mutations by the (CRISPR)/Cas9-based platform was only applied in CLL cell lines such as MEC1 cells. These works used (CRISPR)/Cas9-edited cell line-injected NSG mice for *in vivo* validation of candidate cancer genes of interest and demonstrated the critical roles of high-risk alterations such as del(11q), del(17p), BIRC3, ATM, and TP53 mutations alone or in combination for their biological effects (96, 97), BCR-targeted drug resistance (98), and chimeric antigen receptor (CAR)-T cell therapy responses (99). Thus, future studies on applying (CRISPR)/Cas9-based knock-out and knock-in approaches in the primary CLL patient BM-HSCs or CLL B cells are expected to accelerate the development of novel mouse models of CLL.

To fine-tune human CLL and RT in mice, approaches allowing the engraftment of the entire human immune system are required. Several next generations of humanized mouse strains such as NRG (NOD-*Rag2-IL2rgTm1/*Rj) and NRGS (NRG-SGM3) mice (100), MISTRG mice (expressing human M-CSF, IL-3/GM-CSF, and THPO) (101), and MISTRG-6 (MISTRG with an additional knock-in of the human IL-6 allele) (102) that express human cytokines supporting the engraftment of human HSCs, myeloid cells, and NK cells might enable the generation of CLL mouse models that give a full-blown disease and allow the dissection of the impact of the TME *in vivo*. Combining the (CRISPR)/Cas9 approach with the next-generation humanized mouse strains is expected to facilitate the development of mouse models of CLL and RT for mechanistic and preclinical studies.

## Author contributions

MB: Writing – original draft, Writing – review & editing. S-SC: Writing – original draft, Writing – review & editing.
